# Ag- but Not ZnO-Nanoparticles Disturb the Airway Epithelial Barrier at Subtoxic Concentrations

**DOI:** 10.3390/pharmaceutics15102506

**Published:** 2023-10-21

**Authors:** Helena Moratin, Anna Thöle, Josephine Lang, Totta Ehret Kasemo, Manuel Stöth, Rudolf Hagen, Agmal Scherzad, Stephan Hackenberg

**Affiliations:** Department of Otorhinolaryngology, Plastic, Aesthetic and Reconstructive Head and Neck Surgery, University Hospital Wuerzburg, Josef-Schneider-Strasse 11, 97080 Wuerzburg, Germanyhackenberg_s@ukw.de (S.H.)

**Keywords:** epithelial barrier, nanoparticles, tight junctions, zinc oxide, silver

## Abstract

Inhalation is considered to be the most relevant source of human exposure to nanoparticles (NPs); however, only a few investigations have addressed the influence of exposing the respiratory mucosal barrier to subcytotoxic doses. In the nasal respiratory epithelium, cells of the mucosa represent one of the first contact points of the human organism with airborne NPs. Disruption of the epithelial barrier by harmful materials can lead to inflammation in addition to potential intrinsic toxicity of the particles. The aim of this study was to investigate whether subtoxic concentrations of zinc oxide (ZnO)- and silver (Ag)-NPs have an influence on upper airway barrier integrity. Nasal epithelial cells from 17 donors were cultured at the air–liquid interface and exposed to ZnO- and Ag-NPs. Barrier function, quantified by transepithelial electrical resistance (TEER), decreased after treatment with 10 µg/mL Ag-NPs, but FITC-dextran permeability remained stable and no change in mRNA levels of tight junction proteins and E-cadherin was detected by real-time quantitative PCR (RT-qPCR). The results indicate that subtoxic concentrations of Ag-NPs may already induce damage of the upper airway epithelial barrier in vitro. The lack of similar disruption by ZnO-NPs of similar size suggests a specific effect by Ag-NPs.

## 1. Introduction

Nasal epithelial cells (NECs) are one of the first regions of contact for inhalable particles and pathogens in the human body. Therefore, the integrity of the epithelial barrier is essential for an efficient first line of defense. NECs are part of the respiratory epithelial system, and their biological behavior is representative for the upper airway mucosa. Epithelial cells contribute to both adaptive and innate mucosal immunity by paracrine secretion of cytokines, growth factors, and chemokines that attract T cells. In addition, antimicrobial substances such as lactoferrin or lysozyme are released by these cells [1,2]. The components of the epithelial barrier regulate the passage of liquid and soluble substances through the paracellular space, maintaining homeostasis. Antigens that have penetrated submucosal tissue can trigger specific inflammation.

The junctional complex, which anchors epithelial cells together, consists mostly of apical zonula occludens or tight junctions (TJ), followed by zonula adherens, and the most basal contact site consists of desmosomes [3]. Proteins like tight junctions or zonula occludens (ZO-1, ZO-2), claudins, and occludins are components of the TJ barrier of NECs. E-cadherin is a protein of adherens junctions. Dysfunctionality of TJ is associated with several diseases such as asthma, atopic dermatitis, and inflammatory bowel disease [4,5,6]. TJ disruption in NECs increases exposure of nasal tissues to environmental antigens and may attract inflammatory cells, which can cause tissue inflammation or damage. TJ impairments are also linked to allergic rhinitis and chronic sinusitis. It has been shown that TJ expression patterns in the epithelium of the paranasal mucosa of patients with chronic sinusitis with nasal polyps are patchy, irregular, and with decreased expression of TJ molecules [7]. Harmful effects on nasal TJ have been demonstrated for allergens such as house dust mites or pollens [8,9], cigarette smoke, or airborne pollutants. Such damages may also aggravate pre-existing allergic predispositions [10]. Therefore, it is of great importance to gather more data on the effects of materials, such as nanoparticles (NPs) to which humans are exposed.

Nanotechnology has emerged as one of the key technologies of the 21st century which bears substantial socioeconomical potential [11]. There are numerous innovative efforts to advance medical applications using nanomaterials (NMs). However, the processing of nanoscale materials in everyday products is the primary source of human exposure [12,13]. Silver nanoparticles (Ag-NP) are used in the field of medicine for wound healing materials, antimicrobial agents, and dental implants [14]. Zinc oxide nanoparticles (ZnO-NP) improve rubber flexibility and enhance the vulcanization process. In addition, ZnO-NP processing in the textile, electrical, and food industry is growing. ZnO is already the third most commonly used metal containing NM. According to Liou et al. (2015), workers in a semiconductor manufacturing facility were exposed to NP concentrations ranging from 0.007 to 3.39 mg/m^3^ [15]. Moreover, industrial release of NPs to the environment is expected to increase in the near future [16,17]. Dumont et al. estimated that long-term average concentrations exceed 0.002 ng L^−1^ nano Ag and 1.5 ng L^−1^ nano ZnO concentrations in surface waters across Europe [18].

The deposition patterns of NPs in the respiratory system depend on various factors, such as particle size, shape, surface properties, and the breathing patterns and anatomy of an individual. However, there is evidence that small NPs can reach deep sections of the lung, while larger particles remain in the upper airway [19]. Human exposure to ultrafine particles, i.e., particles <100 nm, which are undesired by-products of, for e.g., combustion processes, leads to chronic cardiovascular and pulmonary morbidity [20]. However, the fate and impact of NPs of similar sizes in the nasal cavity are not yet clear. It is possible that NPs translocate through the olfactory epithelium into the central nervous system [21]. This mechanism of so-called nose-to-brain delivery is used for intranasal drug administration. It allows bypassing the blood-brain barrier, which, in the case of medical treatment, leads to a desired potent and targeted accumulation of the drug in the nervous system [22]. For inhaled NP, the same fate would likely be problematic. In addition, NPs can be absorbed by respiratory epithelium and enter the systemic circulation after infiltration of blood vessels [23], or they can become trapped in mucus, which is eventually swallowed and ends in the digestive tract.

ZnO-NP are known to induce cytotoxic and genotoxic effects in primary human nasal mucosa cells [24], yet so far, there have been few studies on the effects of subcytotoxic doses on the nasal mucosal barrier. More data are urgently needed to evaluate possible aggravation of allergic disposition by airborne exposure to NP. ZnO and Ag are regularly used for industrial manufacturing and processing of consumer products, so that exposure via the airways is possible both for industrial workers and in everyday life [25]. Considering the demonstrated correlations between numerous NPs and human disease and some described mechanisms in the lower respiratory tract, increasing use, and predicted higher future human exposure to Ag- and ZnO-NP, additional data on these abundantly used NPs are needed. Furthermore, it is important to consider concentrations that are not acutely toxic, as such effects are likely to attract attention, whereas subtoxic concentrations may be harmful to an organism due to accumulation or post-exposure effects. NECs are representative of the respiratory epithelium, which extends from the bronchial system to the nose. After inhalation, every section of the respiratory system is potentially exposed to NPs. Hence, NECs are a suitable model for toxicological analysis after exposure to subtoxic NP concentrations. Therefore, the purpose of this study was to investigate whether ZnO- and Ag-NP affect the protective function of the epithelial barrier of the nasal mucosa at subtoxic concentrations.

## 2. Materials and Methods

### 2.1. Isolation and Culture of Human Nasal Mucosa

Human mucosa specimens from 17 patients undergoing functional endoscopic sinus surgery or turbinoplasty were obtained. Specimen from patients with malignant or fungal diseases were excluded. An informed consent statement was signed by all patients prior to surgery and the study was approved by the Ethics Committee of the Medical Faculty of the University of Wuerzburg (vote no. 116/17). The samples were immediately delivered to the cell culture laboratory in 5 mL of saline solution and processed within 60 min. Blood and cartilage residuals were removed by washing the specimens in minimum essential medium (MEM, Sigma-Aldrich, Steinheim, Germany). To prepare the MEM, 500 μL (of 250 µg/mL) Fungizin/amphotericin B, 500 µL (of 50 mg/mL) gentamicin, 5 mL (of 10,000 U/mL) penicillin/streptomycin (Biochrom AG, Berlin, Germany), and 5 mL L-glutamin (200 mM, Sigma-Aldrich) were added to 500 mL of the medium. The tissue was transferred to a 15 mL reaction tube (Greiner Bio-One GmbH, Frickenhausen, Germany) and 9 mL MEM and 100 μL enzyme mix for enzymatic lysis were added. The enzyme mix consisted of 10 mL 1 × phosphate buffered saline (PBS, Roche Diagnostics, Mannheim, Germany) with 1 mg DNAse and 100 mg Protease (both Sigma-Aldrich). To stop cell lysis after 24 h, at 4 °C, 2 mL RPMI medium was added (Biochrom AG), containing 5% fetal calf serum (FCS) (Linaris, Wertheim, Germany), 1% penicillin/streptomycin, 1% 100 mM sodium pyruvate, and 1% of a 100-fold concentration of non-essential amino acids (Biochrom AG). Then, the mechanical cell isolation was carried out using sterile forceps and a scalpel. Then, the cell containing suspension was filtered through a sterile compress into a 50 mL tube (Greiner Bio-One GmbH) and centrifuged at 4 °C and 1000 rpm for 5 min. The supernatant was removed, and the cell pellet was resuspended in 10 mL culture medium. The medium consisted of Airway Epithelial Cell Growth Medium (AEGM, PromoCell GmbH, Heidelberg, Germany) with 1% penicillin/streptomycin (Biochrom AG) and 1% supplements (PromoCell GmbH).

The NECs were further cultured in 6- or 12-well plates (Greiner Bio-One GmbH) or on porous membrane inserts for 12-well plates (Corning^®^ Transwell Polyethylenterephthalat (PET) membrane inserts, 0.4 μm and 12 mm diameter, Corning, Inc., New York, NY, USA). For better cell attachment, wells and inserts were coated with collagen (1 mg/mL, Biochrom AG) in PBS in a 1:1 volume ratio for 30 min at 37 °C. The 12-well plate inserts were coated with 200 µL, and the 6-well plates were coated with 500 µL per well of collagen-PBS solution. The solution was aspirated after 30 min. Then, 500 μL of prepared cell suspension in AEGM from enzymatically degraded tissue was added to each well of the 12-well plates (Greiner Bio-One GmbH, Frickenhausen, Germany) and 1 mL was added to each well of the 6-well plates. In the case of the 12-well plates with inserts, 1 mL of the AEGM was also added to the basal compartment of the well. Cells were cultivated at 37 °C and 5% CO_2_ in the incubator (CO_2_ incubator series CB, Binder GmbH, Tuttlingen, Germany). The AEGM expansion medium was changed every second to third day. When 70% cell confluence in the insert of the 12-well was reached, typically after 10–12 days, it was possible to switch to the air–liquid system by aspirating the inserts apically and performing medium changes in the basal compartment as described. After another period of three days, the experiments were carried out.

### 2.2. Preparation of ZnO- and Ag-Nanoparticles

According to the manufacturer, the used Ag-NP (Sigma Aldrich, product number: 730793, St. Louis, MO, USA) had an average diameter of 20 nm and were present in a suspension with a concentration of 20 µg/mL in aqueous buffer containing sodium citrate as stabilizer. 20 nm of ZnO-NP was purchased as powder (mknano, product number: MKN-ZnO-020, Mississauga, ON, Canada) and 10 mg of ZnO-NP was suspended in 870 µL distillated water. Then, stock solutions of Ag- and ZnO-NP were sonicated for 120 s (Bandelin, Sonopuls HD 60, Berlin, Germany) at a high energy level of 4.2 × 10^5^ kJ/m^3^ using a continuous mode to create a high grade of dispersion. Then, 30 µL of 1.5 mg/mL bovine serum albumin (BSA) dissolved in sterile H_2_O was added to avoid particle agglomeration. Next, 100 µL of 10× concentrated phosphate buffered saline (PBS) was used to create a physiological salt concentration and pH 7.4. A stock solution was generated with supplement-free AEGM. Furthermore, the cell culture medium was changed to supplement-free AEGM 24 h before the start of the experiment in order to avoid falsification of the measured values by the antibacterial penicillin/streptomycin and the supplement mix, which has a supportive effect on cell growth. Cells were exposed in the following volumes: 12 well plastic (500 µL); 6-well plastic (1 mL), and 12-well with inserts, basal compartment (1 mL) AEGM and insert (500 µL).

### 2.3. Characterization of ZnO- and Ag-Nanoparticles

Characterization of ZnO- and Ag-NP has been previously described by our group [26,27]. Briefly, shape, size, and aggregation tendency of particles were evaluated by transmission electron microscopy (TEM) on a Zeiss transmission electron microscope EM 900 (Carl Zeiss, Oberkochen, Germany) at the Division of Electron Microscopy at the University of Wuerzburg Biocenter. The TEM samples were prepared by drop coating the stock suspension on carbon-coated copper grids after sonication and stabilization with BSA. Using a tissue paper, the films on the grids were dried before measurement. The zeta potential and size distribution of the NP aggregates were measured by dynamic light scattering at the Fraunhofer Institute for Silicate Research in Wuerzburg (Malvern Instruments Ltd., Herrenberg, Germany).

### 2.4. MTT Cytotoxicity Assay

To establish a profile of the cytotoxic potential of the used NPs, NECs were incubated with ZnO- and Ag-NP in different doses. The colorimetric tetrazolium (MTT)/formazan assay (here MTT-assay) was used to measure cell viability. Metabolically active cells convert purple tetrazolium salt into yellow formazan. The NECs were cultivated in 12-well plates in AEGM. ZnO-NP, in concentrations from 1 µg/mL to 10 µg/mL, and Ag-NP, in concentrations from 2 µg/mL to 15 µg/mL, were added, and the cells were incubated for 24 h. The highest NP concentration (20 µg/mL) was used as a positive control. The MTT solution was prepared by diluting 12 mg MTT (Sigma-Aldrich) in 12 mL supplement-free AEGM to a final concentration of 1 mg/mL. After removal of the medium, the cells were washed with PBS and 1 mL of diluted MTT solution was added to each well followed by 4 h incubation at 37 °C with 5% CO_2_. Following this period, the MTT solution was replaced by 500 µL Isopropanol (VWR Chemicals, Frontenay-sous-Bois, France) and incubated for 30 min in the dark at room temperature. Wells with the same NP-concentration were pooled and transferred into an Eppendorf tube and centrifuged at 16,000 rpm for 5 min at 4 °C to eliminate particles in the samples. Then, the solution was transferred into a 96-well plate for photometric measurement at 570 nm (ELISA Mikroplate Reader, ELx800, BioTek Instruments GmbH, Bad Friedrichshall, Germany). The results were exported as Excel data files, and values were normalized against the positive control, set to 100%.

### 2.5. Real-Time Quantitative PCR (RT-qPCR) Analysis for Components of the Junctional Complex

The mRNA abundance for genes of the junctional complex was evaluated in NECs after incubation with ZnO- and Ag-NP as described. RT-qPCR was used to measure the mRNA levels of the target proteins E-cadherin, claudin-1, tight junction protein-1, and occludin.

The cells were cultivated in supplement-free AEGM medium in 12-well plates. Then, the Medium was replaced by the same medium containing ZnO-NP (1, 2, 5, 10, and 20 µg/mL) or Ag-NP (2, 5, 10, 15, and 20 µg/mL) and cells were exposed for 24 h. The cells were washed 3 times with PBS and harvested by scraping using a cell scraper (SPL Life Sciences, Gyeonggi-do, Korea). RNA extraction was conducted using the RNeasy Mini Kit (Quiagen GmbH, Hilden, Germany), according to the manufacturer’s instructions. The optical density of 1 µL of the extracted RNA was measured spectrophotometrically (Eppendorf AG, Hamburg, Germany) to determine the RNA concentration. cDNA was synthesized from a total mRNA amount of 50 ng using a SuperScript VILO Mastermix (Applied Biosystems, Life Technologies GmbH, Darmstadt, Germany). The Taq-Man Gene Expression Assay (Thermo Fisher Scientific, Waltham, MA, USA) was used with primers for tight junction protein-1 (TJP1, Assay-ID Hs01551861_m1), occludin (OCLN, Hs001700162_m1), claudin-1 (CDLN1, Hs00221623_m1), and E-cadherin (CDH1, Hs01023895_m1). Glyceraldehyde-3-phosphate dehydrogenase (GAPDH, Hs02758991_g1) was used as the control housekeeping gene. Relative gene expression levels were determined by normalizing the expression of the target gene in relation to that of GAPDH. The ΔΔCT-method was used for relative quantitation of mRNA levels.

### 2.6. Transepithelial Electrical Resistance (TEER) Measurement

TEER was measured to determine the integrity of the epithelial barrier of the NECs before and after expose to Ag- and ZnO-NP. The cells were cultured in a Transwell system with a semipermeable membrane between the apical and basal compartments, as described above. For exposure of the epithelial cells, 1 mL of the ZnO-NP or Ag-NP dispersion was added to the basal compartments of each well and incubated for 24 h at 37 °C. The test was carried out on samples from 11 donors. An epithelial voltometer (EVOM) (World Precision Instruments, Sarasota, FL, USA) generated an alternating current at a frequency of 12.5 Hz and electrical resistance was measured at three positions of the semipermeable membrane of the Transwell bilayer and the average values were calculated. In order to measure the pure resistance of the semipermeable membrane, one well was incubated with collagen-PBS solution and left without cells. The resistances of the Transwell systems with cells were measured in relation to this blank. TEER was measured in untreated cells before exposure to NPs in order to obtain an individual comparison control for each measurement. After calibrating the EVOM to 1000 Ω, the TEER electrode was disinfected in EtOH for 15 min. The electrode was briefly rinsed in sterile H_2_0, and then kept in AEGM for 15 min without additives to adjust the electrode to the pH of the AEGM. Shortly before the start of the measurement, the AEGM of all wells was renewed in order to remove cell degradation products. Subsequently, 1.5 mL medium was added to the basal compartment and 1 mL apically. Means of three measurements per condition and donor were formed. Values were noted, transferred to electronic format and normalized to the cell culture area (1.12 cm^2^), to generate TEER in units of Ω*cm^2^.

### 2.7. Fluorescein Isothiocyanate (FITC)- Dextran Assay

To further analyze the permeability after NP exposure, the FITC-dextran assay was carried out. First, 4 kDa Dextran coupled with fluorescent FITC was added apically to insert cell cultures. Post incubation, the amount of basal fluorescent signal serves as a proxy for permeability. The NECs were seeded in 12-well plates and cultivated. ZnO- and Ag-NP were added, and incubation lasted for 24 h. After removal of the medium, 500 µL of the FITC-dextran solution (product number 60842-46-8, Sigma-Aldrich) was applied to the apical compartment of the insert cell culture and 1 mL of the AEGM was added to the basal compartment, and then the cells were incubated at 37 °C for one hour, protected from light. Subsequently, the FITC-dextran solution was removed from the apical compartment and the solution from the basal compartment of the well was quickly transferred to light-protected 1.5 mL reaction tubes (Safeseal Tube 1.5 mL, product number 72.706.001, Sarstedt AG & Co. KG, Nümbrecht, Germany). To quantify the concentration of FITC-dextran which had permeated the cell layer, the basal solution was analyzed with a fluorescence spectrometer (Infinite^®^200 PRO, TECAN Group, Männedorf, Switzerland) in the neurological department of the University Hospital in Würzburg. For this purpose, the contents of the light-protected tubes of the donors were titrated in 96-well plates (CELLSTAR^®^, 96 Well Cell Culture Plate, product number: 655180, Greiner Bio-One GmbH) according to concentration series. For each sample, three blanks (AEGM without supplements) were added and measured.

### 2.8. Transmission Electron Microscopy (TEM)

Intracellular particle accumulation was investigated by TEM at the Imaging Core Facility, Biocenter, Wuerzburg. Cells were exposed with 2, 5, and 10 µg/mL ZnO-NP and 2, 5, and 10 µg/mL Ag-NP, for 24 h. After trypsinization (0.25% Trypsin-EDTA 1x, Life Technologies Corp. (Gibco), Carlsbad, CA, USA), the cell suspension was transferred to a reaction tube and centrifuged at 270× *g* for 5 min. The supernatant was removed, and the cells were fixed for 45 min at 4 °C in a fresh solution of 0.1 M sodium cacodylate buffer containing 2.5% glutaraldehyde and 2% formaldehyde. The following steps were performed in the core facility. Subsequently, another fixation period for 2 h, at 4 °C, with 2% osmium tetroxide in 50 mM sodium cacodylate (pH 7.2) was carried out after five washing steps of five minutes each with the cocadylate buffer. After rinsing the cells five times with distilled water, they were contrasted overnight using 0.5% uranyl acetate. Finally, the cells were dehydrated in alcohol. After the addition of propylene dioxide and Epon, the specimens were embedded in Epon 812 for 48 h at 60 degrees Celsius, and then sectioned into ultrathin slices. Sections were examined by TEM using an EM 900 (Carl Zeiss, Oberkochen, Germany, funded by the Deutsche Forschungsgemeinschaft (DFG, German Research Foundation)—426173797 (INST 93/1003-1 FUGG)).

### 2.9. Statistical Analysis

The obtained data were statistically analyzed using the GraphPad Prism software 8 (GraphPad Software, Inc., La Jolla, CA, USA). The *t*-test and ANOVA test were implemented to calculate *p*-values, and *p*-values ≤ 0.05 were considered to be statistically significant.

## 3. Results

### 3.1. Nanoparticle Characterization

Characteristics of the NPs used in this study are summarized in Table 1.

### 3.2. Cytotoxicity of ZnO- and Ag-NP

**Figure 1:**
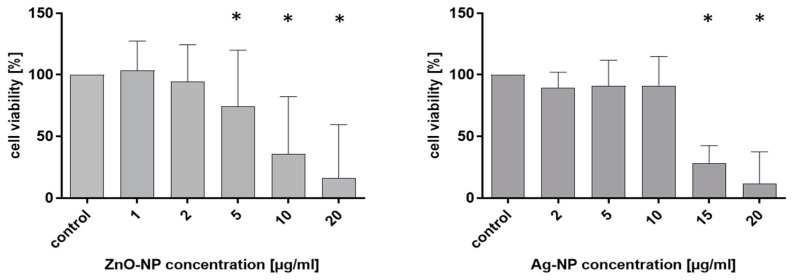
Results of the MTT assay post NP exposure. Cell viability is expressed in (%) compared to untreated cells, which are referred to as the control and the average of all experiments was set as 100%. Nasal epithelial cells (NECs) were incubated with ZnO- and Ag-NP in different concentrations as indicated for 24 h. Dose-dependent cytotoxicity could be observed for both tested NPs, with the reduction of cell viability occurring at higher dosages for Ag-NP compared to ZnO-NP. Data represent the mean ± standard deviation from 10 to 17 donors with 8 replicates each. Statistically significant results are indicated with asterisks.

The MTT assay was used to estimate the cytotoxic effect of ZnO- und Ag-NP. Another aim of the assay was to determine doses in the subcytotoxic range for further experiments. Figure 1 illustrates the measured cell viability of the NECs after 24-h exposure to ZnO- and Ag-NP, expressed as the percentage of viable cells in treated groups as compared with untreated control cells that had a viability of 100%. Both NPs induced a dose-dependent decrease in cell viability, but the effect was especially pronounced for ZnO-NP. Higher doses of Ag- compared to ZnO-NP were necessary to induce a decrease in cell viability. The average cytotoxic effect of ZnO-NP compared to the control was 25.7% after exposure to 5 µg/mL. For Ag, a drastic drop in viability of 75% was seen between 10 and 15 µg/mL Ag, and ~+75% decrease compared to the negative control. Cell viability differed significantly between 5 and 20 µg/mL ZnO- und Ag-NP.

### 3.3. Gene Expression of Junctional Complex Components

**Figure 2:**
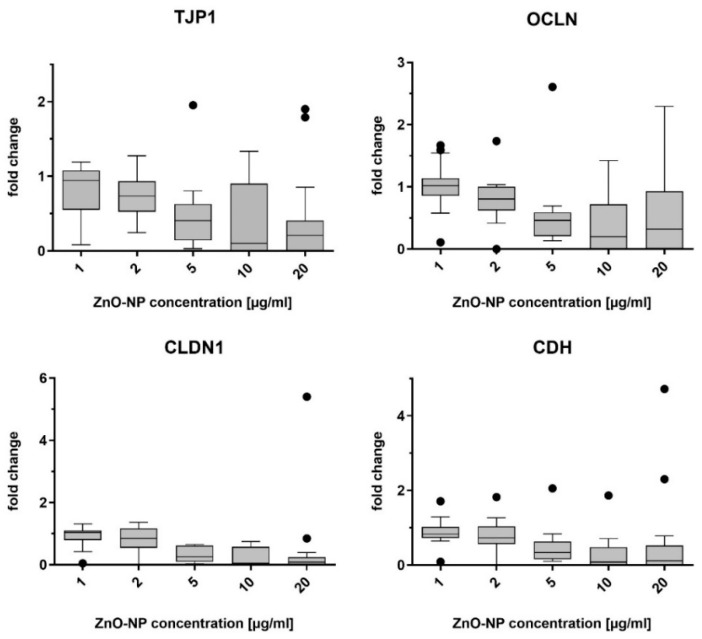
Results of the quantitative real-time PCR of tight junction protein-1 (TJP1), occludin (OCLN), claudin-1 (CLDN1) and E-cadherin (CDH), after incubation of NECs with ZnO-NP at different concentrations for 24 h. Up to a concentration of 2 µg/mL, there were only slight alterations and, from concentrations of 5 µg/mL and higher, there was a distinct decrease in gene expression measurable. Dots in the boxplots indicate statistical outliers (outside 1.5 standard deviations from median). *n* = 11–17.

**Figure 3:**
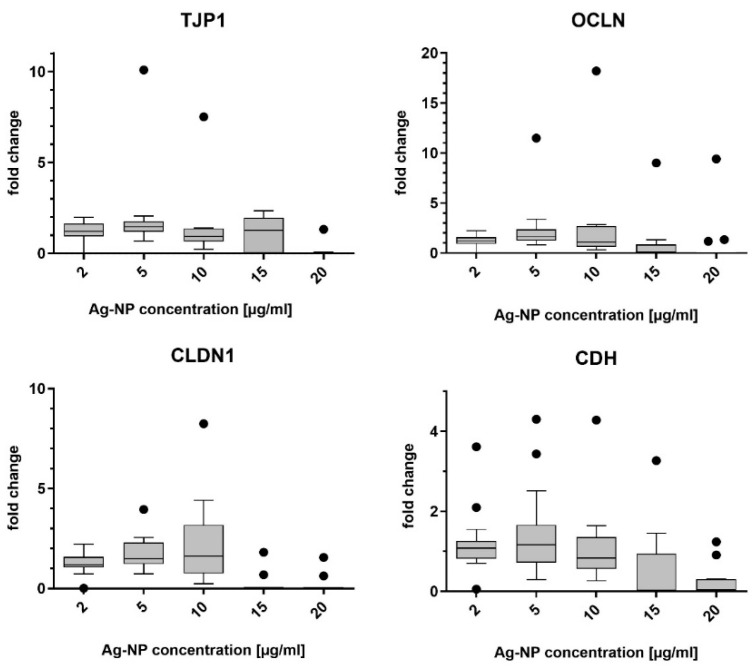
Results of the quantitative real-time PCR of tight junction protein-1 (TJP1), occludin (OCLN), claudin-1 (CLDN1), and E-cadherin (CDH), after incubation of the NECs with Ag-NP at different concentrations for 24 h. Gene expression was stable compared to untreated cells after incubation with 2, 5, and 10 µg/mL. Ag-NP in concentrations of 15 and 20 µg/mL reduced mRNA levels. Dots in the boxplots indicate statistical outliers (outside 1.5 standard deviations from median). *n* = 8–17.

The mRNA levels of the target proteins E-cadherin, claudin-1, tight junction protein-1, and occludin were measured by quantitative real-time PCR. Data are presented as 2^−ΔΔCT^, so that untreated cells (negative control) were set to 1. Figure 2 shows the alterations of gene expression in NECs after incubation with ZnO-NP, and Figure 3 shows the alterations of gene expression in NECs after incubation with Ag-NP. It should be noted that1 and 2 µg/mL ZnO-NP did not significantly change the expression of the four markers. Concentrations of 5 µg/mL, i.e., the lowest cytotoxic concentration, and higher concentrations led to a distinct decrease of the measured mRNA levels.

The one-way ANOVA test was significant after exposure to ZnO-NP for markers tight junction protein-1 and occludin.

Ag-NP in concentrations of 2, 5, and 10 µg/mL did not significantly alter gene expression of the studied markers, while concentrations of 15 and 20 µg/mL caused a decrease in expression.

The one-way ANOVA test was significant for markers tight junction protein-1, E-cadherin, and claudin-1 after incubation with Ag-NP.

### 3.4. Alteration of the TEER

**Figure 4:**
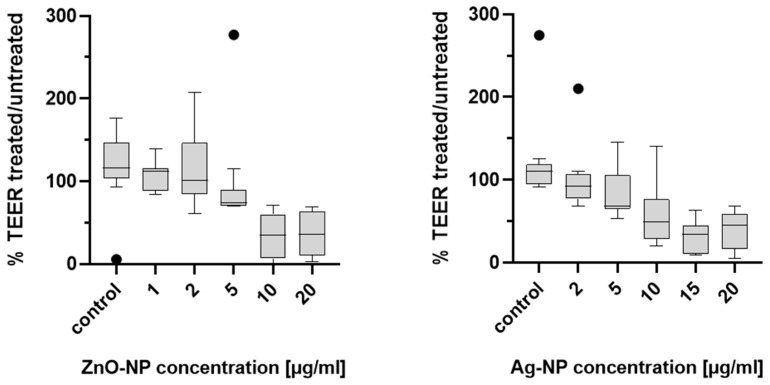
Percentage of TEER values between untreated and NP-treated specimens of 11 donors. Ten and 20 µg/mL ZnO-NP induced markable reduction of TEER. Ag-NP dose-dependently affected TEER with distinct effects from 10 µg/mL. Data is presented with box plots, margins of them illustrate the 25th and 75th percentiles. Whiskers indicate minimal and maximal values. Dots in the boxplots indicate statistical outliers (outside 1.5 standard deviations from median). *n* = 11.

TEER was measured in the same specimen before and after ZnO- and Ag-NP exposures so that the impact on the identical cell population was comprehensible. Resistance values showed a large variance, yet replicable tendencies could be observed for both NPs. Measurements were carried out on samples from 11 different donors. Figure 4 shows the percentage of TEER values of untreated and corresponding treated samples. Overall, a clear drop in TEER after exposure to NPs compared to the untreated specimen was seen from a concentration of 10 µg/mL ZnO-NP (cytotoxic in MTT as-say). Ag-NP dose-dependently affected TEER with a clear effect from 10 µg/mL (subtoxic) and higher.

**Figure 5:**
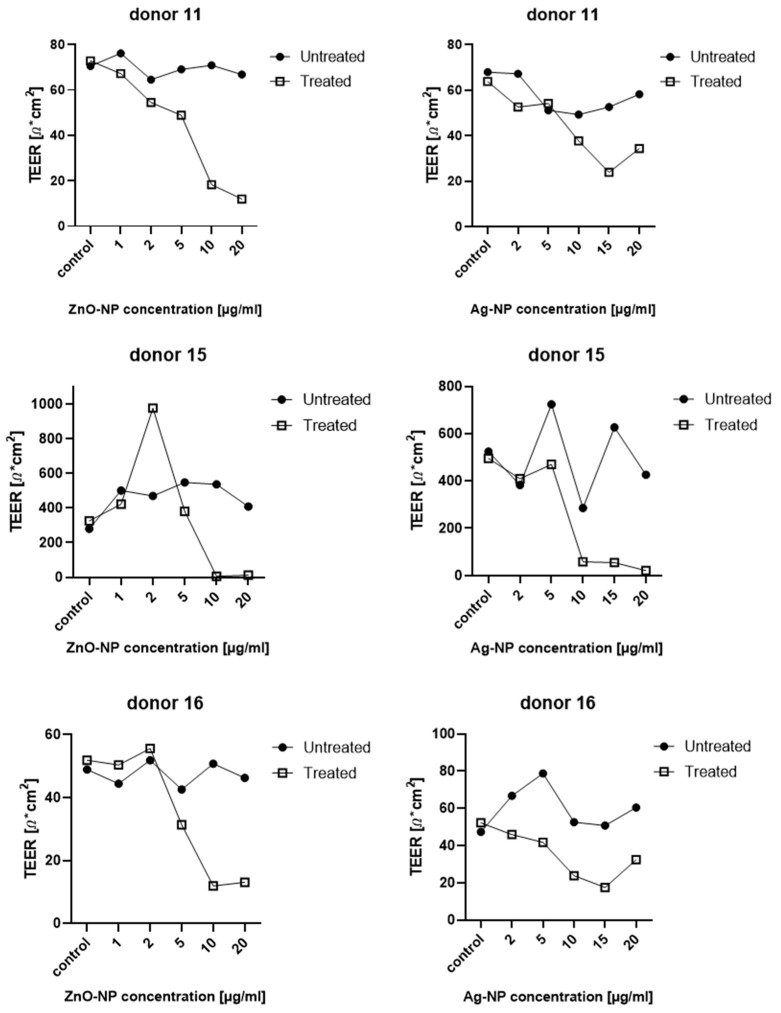
Individual results of TEER measurement for 5 different donors. Individual responses to NP-treatment differed among the donors.

Interindividual differences in susceptibility were high, so that some samples displayed a drop in TEER even at lower doses. Figure 5 demonstrates the individual measured values of five exemplary donors.

### 3.5. Effects on NEC Permeability

**Figure 6:**
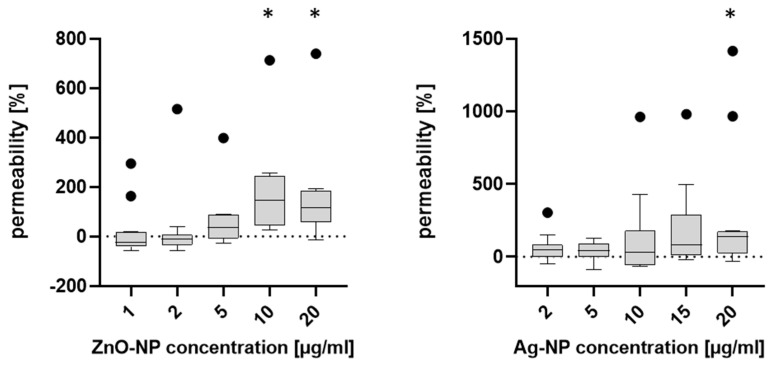
Permeability is expressed as (%) of untreated cells (0%). For ZnO-NP, a dose-dependent increase in permeability could be observed, with 10 and 20 µg/mL inducing significant effects. Ag-NP at a dose of 20 µg/mL significantly altered cell permeability. Asterisks indicate *p* ≤ 0.05 (student’s *t*-test). Data is presented with boxplots, margins of them illustrate the 25th and 75th percentiles. Whiskers indicate minimal and maximal values Dots in the boxplots indicate statistical outliers (outside 1.5 standard deviations from median). *n* = 10–13.

The FITC-dextran assay was used to determine effects on cell permeability after exposure to NPs. Consistent with the TEER results, Figure 6 shows that permeability increased with higher NP concentrations (ZnO: p1µg/mL = 0.7402, p2µg/mL = 0.9298, p5µg/mL = 0.1067, p10µg/mL = 0.0027, p20µg/mL = 0.0024. Ag: p2µg/mL = 0.6494, p5µg/mL = 0.8072, p10µg/mL = 0.8974, p15µg/mL = 0.0540, p20µg/mL = 0.0156; student’s *t*-test).

### 3.6. Intracellular NP Accumulation

**Figure 7:**
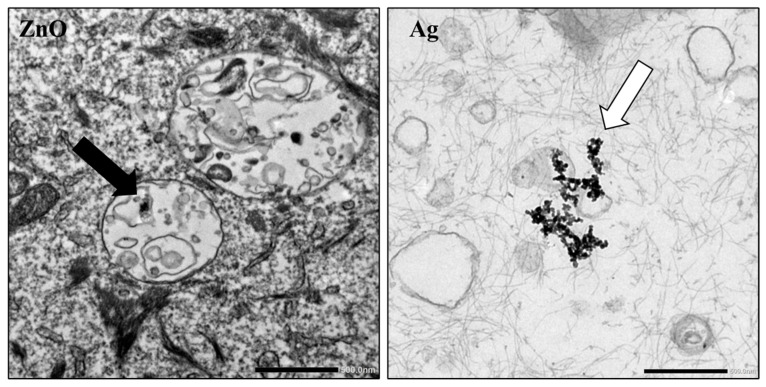
Exemplary transmission electron microscopic images of nasal epithelial cells after exposures to 5 µg/mL ZnO-NP (**left**) and 10 µg/mL Ag-NP (**right**). Small ZnO-NP aggregates are indicated by the black arrow located in the lysosome (scale bar representing 500 nm). The white arrow indicates Ag-NP aggregates in the cytoplasm (scale bar representing 200 nm).

TEM was used to investigate the uptake and accumulation of NPs after exposure of NECs with 2, 5, and 10 µg/mL ZnO-NP and 2, 5, and 10 µg/mL Ag-NP for 24 h. The images show particle agglomerates in the cytoplasm and in cell organelles such as lysosomes, the endoplasmic reticulum, and the cell nucleus. Exemplary images are presented in Figure 7 for 5 µg/mL ZnO- and 10 µg/mL Ag-NP.

## 4. Discussion

Diseases associated with an allergic disposition include atopic dermatitis, asthma, IgE-mediated food allergy, and allergic rhinitis. Since the 1960s, there has been a distinct increase in the prevalence of these medical conditions [28,29]. Questions about the causes are raised and various potential genetic and environmental factors have been discussed. One theory assumes that increased use of antibiotic substances at an early age might play a role [30]. Another hypothesis refers to altered living conditions with increasing urbanization, where the pathophysiological mechanism of the epithelial barrier is thought to be dysfunctionality, supposedly leading to increased infiltration of pathogens, e.g., in the respiratory tract and intestines. Exposure to environmental factors occurring as result of industrialization like diesel exhaust particles (e.g., PM_2.5_ and PM_10_), ozone, cigarette smoke, microplastics, or NPs is considered to have the ability to impede epithelial barrier integrity [31]. Akdis et al. (2021) postulated that a dysfunctional epithelial barrier facilitates the translocation of bacteria to the interepithelial and subepithelial area, where tissue microinflammation is initiated [32]. Moreover, the uptake of allergens and exogenous particles can be enhanced, leading to increased activation of mast cells and nerve fibers [33], possibly causing local inflammation.

The nasal epithelium is the first region of contact for inhaled pathogens, airborne particles, and allergens. One essential part of an intact epithelial barrier are TJ, which control paracellular transport and maintain transepithelial homeostasis [34]. Association between impaired TJ functionality and different diseases has been reported. For example, patients suffering from asthma displayed disruption of TJ in bronchial biopsies [5]. Furthermore, TJ dysfunction has been described in context with allergic rhinitis and even with the distinctions between the subtypes of chronic rhinosinusitis with and without nasal polyps [7,35]. Various exogenous substances have been studied for their potential to damage the epithelial barrier. Intranasal application of 10 µg diesel exhaust particles in 20 µL vehicle solution decreased the signal of nasal epithelial ZO-1 and increased the occurrence of allergy-related symptoms in ragweed-pollen-sensitized mice after co-administration of intranasal ragweed [36]. Exposure to naturally occurring pathogens like *Alternaria alternata* [37], yet also, anthropogenic factors like particulate matter (2.5 µm) are harmful [38] and assumed to partly cause effects by damaging the epithelial barrier.

There are many studies that have addressed the cytotoxic and genotoxic properties of NPs, including the mechanisms of action. However, with increasing risk of repetitive low-dose exposure via inhalation, more information is needed on the effects of NPs on upper airway epithelial cells as the first region of contact in the human body. Therefore, human nasal epithelial cells were chosen as a model in this study and effects on the barrier function were evaluated after exposure to ZnO- and Ag-NP. Cell cultivation at the air–liquid interface is an eligible method to recapitulate airway epithelia biology as the transcriptional profile closely resembles that of in vivo airway epithelia [39]. On the one hand, the advantage of primary cells is the comparability of features of the original tissue, including genetic makeup and function, which makes them more biologically relevant and representative of the in vivo conditions. This enables a more realistic response to pharmacological agents and toxic substances. On the other hand, the availability of primary cells is limited, and they naturally exhibit high donor variability.

TEM images show NP agglomerates present intracellularly. Thus, it can be assumed that the Ag-NP agglomerate under standard cell culture conditions and are internalized by the cells. In this study, these agglomerates could be illustrated using TEM. However, as described by Smith et al. (2018), the toxicological properties are presumably also mediated by dissolved silver ions [40]. The results of this study show that exposure of NECs to 1 and 2 µg/mL ZnO-NP and 2, 5 and 10 µg/mL Ag-NP did not significantly alter cell viability, and therefore these concentrations were considered subcytotoxic. This also agrees with data from previous studies by the working group [24]. To investigate the pathomechanisms of NP exposure at the molecular level, the gene expressions of different components of epithelial intercellular junctions were measured. Tight junction protein-1, which is also called zonula occludens-1 (TJP1/ZO-1), occluding, and claudin are integral elements of TJ. E-cadherin is an adherens junction protein [41]. Subcytotoxic concentrations of ZnO- and Ag-NP did not reduce gene expression in this study. However, other investigations of protein levels and localization (in immunofluorescence assays) as opposed to mRNA have revealed that ZnO-NP can impair endothelial paracellular barriers in mice after inhalation exposure by decreasing the expression level of some proteins and changing the distribution of tight and adherens junction proteins [42]. Imai et al. (2017) showed that the exposure side (apical versus basolateral surface of the epithelial layer) plays an important role in terms of internalization and transcellular transport of Ag-NP [43]. TJ are located near the apical portion of the intercellular space. Therefore, an interaction of NPs with TJ after basolateral exposure seems less likely. Another possibility would be that no change in gene expression on apically located structures is induced in cells by basolateral exposure. However, it must be taken into consideration that the quantification of gene expression patterns by RT-PCR only allows a snapshot of the intracellular mechanisms. Methods assessing protein expression patterns like Western blot or immunofluorescence could provide more information.

Maintaining TEER is a characteristic function of an intact epithelial barrier. A variety of key mechanistic pathways can lead to changes in TEER and increased permeability in cells. In particular, oxidative stress can lead to disruption of tight junctions, which is considered to be a main factor of NP-associated toxicity [44]. Moreover, inflammatory stimuli like tumor necrosis factor-α (TNFα) and interleukins have the potential to influence barrier integrity [45,46]. De Planque et al. (2011) proposed that NP–lipid interactions alone can compromise the barrier function of the plasma membrane [47]. This can lead to an increase in paracellular permeability and a decrease in TEER [48]. However, the specific effect of NPs on the integrity of the epithelial barrier also depends significantly on particle-specific characteristics such as their size, charge, and surface properties. ZnO- and Ag-NP exposure has been shown to reduce the resistance in a dose-dependent manner. As expected, when working with primary cells, interindividual TEER variability was high. However, tendencies of distinct cellular responses after NP exposure were detected. Concentrations of ZnO-NP of 5 µg/mL and higher reduced TEER. This correlated with the cytotoxic concentration and may be a consequence of the cytotoxic effect. It is also possible that more fine-tuned titration of particles would reveal a barrier disruption at lower concentrations than the cytotoxic effect, similar to the presented Ag-NP results. Ag-NP have been shown to affect TEER already at subtoxic doses with effects at 10 µg/mL, which did not induce cytotoxicity. The impairment of the barrier integrity could be a result of inflammatory stimuli. Hackenberg et al. (2011) found an increase of cytokine release after exposition with Ag-NP [27]. The results of the TEER measurement are in accordance with the study of Xu et al. (2015) who showed that exposure to 10 µg/mL Ag-NP reduced TEER in a rat blood-brain barrier model. Moreover, ZO-1 expression decreased in the immunostaining in that study [49]. The presented results indicate that subcytotoxic concentrations of Ag-NP have an influence on the cells’ capabilities of maintaining barrier integrity. Interestingly, this did not correlate with the results of the FITC-dextran assay, where permeability increased only after 15 and 20 µg/mL Ag-NP. Apparently, the damage to the epithelial barrier is not severe enough to allow passage for large and branched molecules such as fluorescein isothiocyanate-dextran (FD4), which has an average molecular weight from 3000 to 5000 Dalton. Soluble substances can cross epithelial cell layers via transcellular or paracellular pathways. The transcellular pathway implies a crossing of the apical and the basolateral cell membrane, whereas the paracellular pathway means a passage through the tight junctions. Conventional TEER measurements cannot discriminate para- and transcellular resistance [50]. Since no difference of gene expression of TJ proteins could be detected in the actual study, a change in transcellular transport could be the cause of the TEER decrease after Ag-NP exposure.

Studies have shown that baseline TEER values and mRNA expression of TJ genes in patients with allergic rhinitis (AR) or chronic rhinosinusitis with nasal polyps differs significantly from healthy donors [51]. It is perceivable, that the effects of NPs are overshadowed by donor variability caused by such pre-conditions. Considering the data on TJ proteins in mouse studies, investigations of TJ proteins as opposed to mRNA in NECs in vitro may provide a stronger signal, which is detectable in spite of donor variability. Pre-existing conditions of the nasal mucosa might, therefore, cause a higher susceptibility to exogenous allergens or pathogens. For example, Ogi et al. (2021) demonstrated that patients with house dust mite (HDM) allergic rhinitis were more sensitive to the HDM-associated allergen *Dermatophagoides pteronyssinus 1* than non-AR patients. This resulted in an increased impairment of mucosal function and IL-6 secretion [52]. Also, environmental factors (rural vs. urban living conditions), smoking status, or a previous application of cortisone-containing nasal spray could play a role and should be taken into consideration for further investigations.

In summary, on the one hand, exposure of NECs to subcytotoxic doses of ZnO-NP showed no effects on the epithelial barrier function in the experimental setup of this study. On the other hand, subcytotoxic Ag-NP at a concentration of 10 µg/mL induced a reduction in TEER, whereas permeability did not increase in the FITC-dextran assay. No differences in the gene expression of TJ proteins and E-cadherin were measurable. This could be explained by an incubation period that was too short. In addition, PCR studies provide limited information, since only actual changes in protein expression have biological relevance. When working with nanomaterials, it must be taken into consideration that general statements on toxic properties can hardly be made. The substances often differ significantly in terms of physical and chemical characteristics that are decisive for their toxicological profile. However, this study indicates that Ag-NP can have an impact on the barrier function of nasal mucosa cells. Multidimensional nasal mucosa models should be considered for further studies in order to enable a more realistic experimental setup and an approximation to in vivo conditions.

## 5. Conclusions

The results of this study provide information about the interaction of environmentally relevant NPs with NECs as representatives of the respiratory epithelium. Subtoxic exposure conditions resulted in reduced TEER values, suggesting that Ag-NP may influence the integrity of the epithelial barrier.

## Figures and Tables

**Table 1:** Particle characteristics of ZnO- and Ag-NP.

	ZnO-NP	Ag-NP
Zeta potential	−11.2 mV	−13.6 mV
Mean diameter	20–30 nm	46 ± 21 nm (mean ± SE)
Hydrodynamic diameter	67.06 nm	404 nm

## Data Availability

The data that support the findings of this study are available from the corresponding author upon reasonable request.
